# Oligometastatic Prostate Cancer

**DOI:** 10.1007/s11864-016-0439-8

**Published:** 2016-10-27

**Authors:** Daniel J. Stevens, Prasanna Sooriakumaran

**Affiliations:** Nuffield Department of Surgical Sciences, John Radcliffe Hospital, University of Oxford, Room 6607, Level 6, Headington, Oxford, OX3 9DU UK

**Keywords:** Prostate cancer, Oligometastatic

## Abstract

The mainstay of treatment for men with three or fewer non-castrate metastatic lesions outside of the prostate remains morbid palliative androgen deprivation therapy. We believe there is now a significant body of retrospective literature to suggest a survival benefit if these men have radical treatment to their primary tumour alongside ‘metastasis-directed therapy’ to the metastatic deposits. However, this regimen should be reserved to high-volume centres with quality assurance programmes and excellent outcomes. Patients should be made clear as to the uncertainty of benefit for this multi-site treatment strategy, and we await the publication of randomised controlled trials reporting in the next 5 years.

## Introduction

Prostate cancer is the commonest cancer and the most frequent cause of cancer death in Western men [1]. The median survival for men presenting with metastatic disease is 42.1 months [2•]. Currently accepted treatment consists of androgen deprivation therapy (ADT) followed by chemotherapy and novel agents once the ADT is no longer effective. Treating metastatic disease is estimated to cost healthcare systems US$20,000 per man [3]. Oligometastatic disease can be defined as the development of three or fewer non-castrate lesions outside of the primary tumour [4••]. These can be bone metastases or in the soft tissue. The concept of oligometastatic disease for all cancers is based around a stepwise progression whereby a cancer initially metastasises in a limited way, before acquiring widespread metastatic behaviour [5]. In a range of solid tumours [6–8], it is hypothesised that this intermediate step represents a different biology compared to extensive metastatic disease, and therefore, it represents an opportunity to influence the rate of progression, and possibly even cure the patient. It is thought that in oligometastatic states, the true metastatic growth potential is limited. This may be secondary to tumour microenvironments in the primary lesion remaining sufficiently hospitable that evolutionary clonal pressure is low; therefore, cancer cells that slough off the primary do not have the properties necessary to survive the circulation and invade target organ sites effectively [9•]. This is contrasted with systemic metastatic disease where the primary tumour has created many undifferentiated aggressive clones that actively migrate out of the primary tumour and have the characteristics to survive and invade target organ sites. The clinical implication of oligometastatic disease is that treatment of metastases alongside the primary tumour (if not already treated) could result in long-term survival or cure [10, 11]. Treatment of oligometastatic disease may also decrease disease-related morbidity and reduce overall tumour burden. However, without randomised controlled trials, it is very difficult to know if the treatment of oligometastatic disease helps the patient. The current literature in all cancer types is limited by using varying definitions of oligometastasis, with a variety of endpoints and at different levels of evidence [9•]. With regard to prostate cancer, while standard treatment remains long-term palliative androgen deprivation treatment, there is emerging data that treatment of the primary with stereotactic body radiotherapy (SBRT) or even radical prostatectomy may impact survival, slow symptomatic disease progression, and reduce the need for palliative surgical intervention [12–15]. In this review, we evaluate the three main treatment approaches for oligometastatic prostate cancer—systemic therapy, treatment of the primary tumour and metastasis-directed therapy.

## Systemic Therapy

The standard treatment options and guidelines for prostate cancer (PCa) patients diagnosed with metastatic progression following primary treatment have remained largely unchanged over the last decade [16••]. Androgen deprivation therapy (ADT) remains the recommended treatment for men with any metastatic disease [16••]. There is no level 1 evidence for or against a specific form of ADT, with orchidectomy, LHRH analogue or antagonist all being acceptable options. The exception remains patients with impending spinal cord compression for whom orchidectomy or LHRH antagonist are preferred [16••]. ADT can be given alone or in combination with another anti-androgen such as bicalutamide. Combined androgen blockade appears to show a small advantage in systematic reviews when compared with monotherapy over more than 5 years [17, 18]. However, this advantage must be weighed against the increased side effects of combined treatment. ADT can also be given continuously or intermittently. Intermittent androgen deprivation may offer better bone protection and a protective effect against metabolic syndrome, but the evidence is not conclusive and most trials comparing the two have several limitations. A recent review concluded that there is a small improvement in quality of life, but was unable to prove non-inferiority [19]. Recently, data has emerged from the STAMPEDE, CHAARTED and GETUG-15 trials that support the use of docetaxel as a first-line treatment alongside ADT. It has been shown to improve survival and delay the onset of castrate resistance [20••]. The STAMPEDE trial is also evaluating the addition of enzalutamide to ADT in newly diagnosed metastatic patients and the addition of both enzalutamide and abiraterone to ADT in the same patient population [21]. The results of the trial may lead to further combination therapies as first-line treatment. Despite a multitude of trials and being the preferred treatment for decades, there is no evidence regarding the outcomes of the sub-set of men who have oligometastatic disease as opposed to the widespread systemic (polymetastatic) cohort. In men with widespread systemic metastases, there is a very real risk of symptomatic bone pain, renal failure, anaemia, pathological fractures and spinal compression. The early implementation of ADT has been shown to reduce these risks [22]. However, in the setting of oligometastatic disease, where the likelihood of these clinical manifestations is much lower, it may be that alternative treatments are more appropriate. This is particularly significant when the morbidity and reduction in quality of life that ADT causes is taken into account. Despite the evolving thinking in oligometastatic disease, ADT + docetaxel remains the standard of care in most centres.

## Treating the Primary Site

Decreasing primary tumour burden, through cytoreductive or radical surgery, radiotherapy or both together has been shown to improve survival in a number of metastatic malignancies. Cytoreductive surgery has shown survival benefit in colon [23], breast [24] and ovarian cancer [25]. Radical surgery to remove the primary in the setting of metastases has been associated with improved survival in glioblastoma [26], renal cell carcinoma [27] and colorectal cancer [28]. EORTC and Southwest Oncology Group studies have demonstrated that nephrectomy plus systemic therapy offers a 13–36 % improvement in survival when compared to systemic therapy alone [27, 29]. Similarly, a meta-analysis of 6885 women with advanced ovarian carcinoma found a median survival of 33.9 months in those treated with >75 % maximal cytoreductive surgery versus 22.7 months for those with ≤25 % cytoreduction [25]. The exact mechanism underlying these results is not clear but there are a number of biologically plausible explanations. The ‘soil and seed’ hypothesis postulates that tumour cells require an appropriate microenvironment in which to engraft at the site of metastasis. A number of studies have demonstrated a role for the primary tumour in ‘priming’ the metastatic niche through the secretion of membrane-bound vesicles such as exosomes which can transfer proteins and nucleic acids to distant sites in advance of the engraftment of circulating tumour cells [30, 31]. Genetic interplay between primary and distant sites may play a role in the development of metastatic prostate cancer. Interruption of this cycle by treating radically or in a cytoreductive fashion may alter tumour biology and result in depressed growth and/or limit the growth of future metastatic sites.

There are currently no reported prospective data regarding a survival benefit for patients with metastatic prostate cancer who subsequently undergo treatment of the primary tumour. However, a number of retrospective studies support this strategy. A sub-group analysis of the SWOG 8894 trial found that 1286 men with metastatic prostate cancer showed a reduced risk of death for those who had previously undergone radical prostatectomy compared to those that had not [32]. Recent observational cohort studies from the US SEER database and the Munich Cancer Registry found that men with metastatic prostate cancer treated with radical therapy had higher 5-year survival than those treated with systemic therapy alone [33•, 34•]. Sooriakumaran et al. recently showed that at least 1206 men in Sweden have been treated with initial radical therapy (surgery or radiotherapy) for likely metastatic or micrometastatic prostate cancer from 1996 to 2010 [35], and on further interrogation of 18,352 cases found that men who underwent initial ADT without radical therapy were approximately three times more likely to die of prostate cancer than those that had radical therapy (manuscript in preparation). Very recently published data from the Prostate Cancer data Base Sweden (PCBaSe) further supports these results by showing that men with very high-risk prostate cancer for whom radical therapy has traditionally been seen as ineffective, have substantially lower prostate cancer and all-cause mortality when treated in units with the highest exposure to radical treatment (surgery or radiotherapy) [36]. Increasingly, the evidence is suggesting patients with high-risk and metastatic disease may benefit from radical or cytoreductive local therapy where patient selection is appropriate and the unit they are treated in sufficiently experienced.

## Treating the Metastases

Metastasis-directed therapy (MDT) is usually reserved for a sub-set of patients with a limited number of metastases (typically three or fewer) and therefore represents those patients defined as oligometastatic. The aim is to control the cancer and slow down any further metastasis while avoiding or delaying the toxicity associated with the use of systemic therapies [37]. This is particularly true considering the detrimental effect of ADT on general health and quality of life. MDT is commonly offered in the setting of oligometastatic colorectal, sarcoma and renal cell carcinoma [7, 38, 39]. Although no randomised controlled trials are available comparing MDT to no treatment, MDT is routinely offered based on the results of promising case series and large patient registries [40]. The literature in this setting refers to metachronous rather than synchronous oligometastases, i.e. metastases developed some time after initial radical therapy—in effect a treatment failure or an initial mis-staging. It is uncertain whether results would be comparable in the setting of oligometastases being detected at the time of the first diagnosis. Ost et al. carried out a systematic review in 2015 that examined seven studies reporting on patients who had metachronous metastases with a “controlled” primary (defined as previous curative treatment to the primary PCa) who received MDT via surgical metastasectomy or non-palliative radiotherapy [4••]. Overall, 51 % of patients were progression free at 1–3 years after MDT suggesting a promising approach for MDT. However, the low level of evidence and the small number of studies do not allow extrapolation to standard of care. A further analysis from Ost et al. in 2016 pooled the individual patient data obtained from different institutions treating metachronous oligometastatic PCa with stereotactic body radiotherapy [41]. One hundred nineteen patients with 163 metastases were treated with a median progression-free survival of 21 months. A dose response was demonstrated with higher radiation doses leading to better rates of survival. The implication from these results is that localised forms of cancer treatment might be sufficient in these patients in order to delay systemic treatments until progression to widespread metastases. There is no evidence that delaying ADT in well-informed asymptomatic men with metastatic prostate cancer impacts survival, and the EAU recommends this strategy where appropriate [16••]. MDT for prostate cancer recurrence appears promising and may represent a paradigm shift. However, its role in synchronous oligometastases is not clear, especially regarding how this would be delivered alongside radical/cytoreductive therapy for the primary tumour. The results of prospective comparative or randomised trials are awaited before recommending changes to standard of care (Table 1).

**Table 1:** Novel treatment strategies for oligometastatic prostate cancer

Synchronous disease	
Radical prostatectomy or prostate radiotherapy, or	
Stereotactic body radiotherapy to the sites of oligometastases, or	
Both of the above	
Metachronous disease	
Stereotactic body radiotherapy to the sites of oligometastases, or	
Salvage prostate-directed therapies, or	
Both of the above	

In both settings, patients should be offered clinical trials where these are available, as none of the above treatment strategies are standard of care

## Future Work

In order for clinicians to safely and ethically offer patients surgery and/or radiotherapy for oligometastatic prostate cancer, it is important to collect high quality evidence, ideally from randomised controlled trials. The Surveillance or metastasis-directed Therapy for OligoMetastatic Prostate cancer recurrence (STOMP) trial is a phase II RCT being run from Belgium that will report in 2017 [42]. In patients with metachronous oligometastatic disease it aims to compare MDT with active clinical surveillance. The hypothesis here is that MDT will prolong the time until palliative ADT is required. The Conventional care or Radioablation in the treatment of Extracranial metastases (CORE) trial running at the Royal Marsden Hospital (London, UK) is examining whether the addition of stereotactic ablative SBRT delivered to metachronous oligometastases can increase progression-free survival compared to standard care [43]. However, it will not report until 2021. MD Anderson Cancer Centre is running a trial comparing best systemic therapy (BST) alone versus BST plus radical prostatectomy or radical radiotherapy directed at the primary in synchronous metastatic disease, which includes polymetastatic disease also [44]. The primary outcome is progression-free survival and it is due to report in 2018. Testing Radical prostatectomy in men with oligometastatic prostate cancer that has spread to the bone (TRoMbone) is a pilot RCT (Oxford, UK) that will randomise men with synchronous oligometastatic disease to treatment as usual versus treatment as usual plus radical prostatectomy [45]. It will recruit 50 patients and report in 2018. Finally, the g-RAMPP trial based at the Martini-Klinik in Germany has a similar protocol to TRoMbone but is attempting to recruit directly to a full trial [46]. Its primary endpoint is prostate cancer-specific survival and needs to enrol 452 patients with a planned report date of 2025.

Clearly, in the next few years, the evidence supporting how to best treat oligometastatic disease will be strongly progressed and allow clinicians to better advise their patients. Of note, the new imaging technology, for example, PSMA-targeted PET agents, that are being rapidly adopted in some parts of the world is likely to change the clinical workflow in terms of the way patients are staged. We await with interest how these more accurate imaging strategies will impact on the numbers of patients being diagnosed with oligometastatic disease, and how it changes treatment practices.

## References

[1] Siegel R, Ma J, Zou Z, Jemal A (2014). Cancer statistics, 2014. CA Cancer J Clin.

[2.•] James N, Spears M, Clarke N, Dearnaley D, De Bono J, Gale J (2015). Survival with newly diagnosed metastatic prostate cancer in the “Docetaxel Era”: Data from 917 Patients in the control arm of the STAMPEDE trial (MRC PR08, CRUK/06/019). Eur Urol.

[3] Heijnsdijk E, de Carvalho T, Auvinen A, Zappa M, Nelen V, Kwiatkowski M (2014). Cost-effectiveness of prostate cancer screening: a simulation study based on ERSPC Data. JNCI J Nat Cancer Inst.

[4.••] Ost P, Bossi A, Decaestecker K, De Meerleer G, Giannarini G, Karnes R (2015). Metastasis-directed therapy of regional and distant recurrences after curative treatment of prostate cancer: a systematic review of the literature. Eur Urol.

[5] Khoo V (2016). Is there another bite of the cherry? The case for radical local therapy for oligometastatic disease in prostate cancer. Eur Urol.

[6] García-Yuste M, Cassivi S, Paleru C (2010). The number of pulmonary metastases: influence on practice and outcome. J Thorac Oncol.

[7] Spelt L, Andersson B, Nilsson J, Andersson R (2012). Prognostic models for outcome following liver resection for colorectal cancer metastases: a systematic review. Eur J Surg Oncol (EJSO).

[8] Salah S, Watanabe K, Welter S, Park J, Park J, Zabaleta J (2012). Colorectal cancer pulmonary oligometastases: pooled analysis and construction of a clinical lung metastasectomy prognostic model. Ann Oncol.

[9.•] Reyes D, Pienta K (2015). The biology and treatment of oligometastatic cancer. Oncotarget.

[10] Weichselbaum RHellman S. Oligometastases revisited. Nat Rev Clin Oncol. 2011. 10.1038/nrclinonc.2011.44 21423255

[11] MacDermed D, Weichselbaum R, Salama J (2008). A rationale for the targeted treatment of oligometastases with radiotherapy. J Surg Oncol.

[12] Wiegand L, Hernandez M, Pisters L, Spiess P (2010). Surgical management of lymph-node-positive prostate cancer: improves symptomatic control. BJU Int.

[13] Won A, Gurney H, Marx G, De Souza P, Patel M (2013). Primary treatment of the prostate improves local palliation in men who ultimately develop castrate-resistant prostate cancer. BJU Int.

[14] Schweizer M, Zhou X, Wang H, Yang T, Shaukat F, Partin A (2013). Metastasis-free survival is associated with overall survival in men with PSA-recurrent prostate cancer treated with deferred androgen deprivation therapy. Ann Oncol.

[15] Ost P, Decaestecker K, Lambert B, Fonteyne V, Delrue L, Lumen N (2014). Prognostic factors influencing prostate cancer-specific survival in non-castrate patients with metastatic prostate cancer. Prostate.

[16.••] Eidenreich A, Bastian P, Bellmunt J, Bolla M, Joniau S, van der Kwast T (2014). EAU guidelines on prostate cancer. Part II: treatment of advanced, relapsing, and castration-resistant prostate cancer. Eur Urol.

[17] Maximum androgen blockade in advanced prostate cancer: an overview of the randomised trials. Lancet. 2000;355(9214):1491–1498. 10801170

[18] Schmitt B, Bennett C, Seidenfield J, Samson D, Wilt T (2000). Maximal androgen blockade for advanced prostate cancer. Cochrane Database Syst Rev.

[19] Hussain M, Tangen C, Berry D, Higano C, Crawford E, Liu G (2013). Intermittent versus continuous androgen deprivation in prostate cancer. N Engl J Med.

[20.••] Vale C, Burdett S, Rydzewska L, Albiges L, Clarke N, Fisher D (2016). Addition of docetaxel or bisphosphonates to standard of care in men with localised or metastatic, hormone-sensitive prostate cancer: a systematic review and meta-analyses of aggregate data. Lancet Oncol.

[21] [Internet]. 2016 [cited 15 September 2016]. Available from: http://www.stampedetrial.org/87548/87552/STAMPEDE_Protocol_v14_clean.pdf

[22] Immediate versus deferred treatment for advanced prostatic cancer: initial results of the Medical Research Council trial. Br J Urol. 1997;79(2):235–246. 10.1046/j.1464-410x.1997.d01-6840.x 9052476

[23] Glehen O, Mohamed F, Gilly F (2004). Peritoneal carcinomatosis from digestive tract cancer: new management by cytoreductive surgery and intraperitoneal chemohyperthermia. Lancet Oncol.

[24] Polychemotherapy for early breast cancer: an overview of the randomised trials. Lancet. 1998;352(9132):930–942. 9752815

[25] Bristow R (2002). Survival effect of maximal cytoreductive surgery for advanced ovarian carcinoma during the platinum era: a meta-analysis. J Clin Oncol.

[26] Nitta TSato K (1995). Prognostic implications of the extent of surgical resection in patients with intracranial malignant gliomas. Cancer.

[27] Mickisch G, Garin A, van Poppel H, de Prijck L, Sylvester R (2001). Radical nephrectomy plus interferon-alfa-based immunotherapy compared with interferon alfa alone in metastatic renal-cell carcinoma: a randomised trial. Lancet.

[28] Temple L (2004). Use of surgery among elderly patients with stage IV colorectal cancer. J Clin Oncol.

[29] Flanigan R, Salmon S, Blumenstein B, Bearman S, Roy V, McGrath P (2001). Nephrectomy followed by interferon alfa-2b compared with interferon alfa-2b alone for metastatic renal-cell cancer. N Engl J Med.

[30] Costa-Silva B, Aiello N, Ocean A, Singh S, Zhang H, Thakur B (2015). Pancreatic cancer exosomes initiate pre-metastatic niche formation in the liver. Nat Cell Biol.

[31] Peinado H, Alečković M, Lavotshkin S, Matei I, Costa-Silva B, Moreno-Bueno G (2012). Melanoma exosomes educate bone marrow progenitor cells toward a pro-metastatic phenotype through MET. Nat Med.

[32] Thompson I, Tangen C, Basler J, Crawford E. Impact of previous local treatment for prostate cancer on subsequent metastatic disease. J Urol. 2002;1008–12. 10.1016/S0022-5347(05)64562-4 12187210

[33.•] Culp S, Schellhammer P, Williams M (2014). Might men diagnosed with metastatic prostate cancer benefit from definitive treatment of the primary tumor? A SEER-based study. Eur Urol.

[34.•] Gratzke C, Engel J, Stief C (2014). Role of radical prostatectomy in metastatic prostate cancer: data from the Munich Cancer Registry. European Urology.

[35] Sooriakumaran P, Nyberg T, Akre O, Haendler L, Heus I, Olsson M (2014). Comparative effectiveness of radical prostatectomy and radiotherapy in prostate cancer: observational study of mortality outcomes. BMJ.

[36] Stattin P, Sandin F, Thomsen F, Garmo H, Robinson D, Lissbrant I et al. Association of radical local treatment with mortality in men with very high-risk prostate cancer: a semiecologic, nationwide, population-based study. European Urology. 2016. 10.1016/j.eururo.2016.07.023 27481175

[37] Hellman S, Weichselbaum RR (1995). Oligometastases. J Clin Oncol.

[38] Kim D, Karam J, Wood C (2014). Role of metastasectomy for metastatic renal cell carcinoma in the era of targeted therapy. World J Urol.

[39] Tree A, Khoo V, Eeles R, Ahmed M, Dearnaley D, Hawkins M (2013). Stereotactic body radiotherapy for oligometastases. Lancet Oncol.

[40] Treasure T, Milošević M, Fiorentino F, Macbeth F (2014). Pulmonary metastasectomy: what is the practice and where is the evidence for effectiveness?. Thorax.

[41] Ost P, Jereczek-Fossa B, As N, Zilli T, Muacevic A, Olivier K (2016). Progression-free survival following stereotactic body radiotherapy for oligometastatic prostate cancer treatment-naive recurrence: a multi-institutional analysis. Eur Urol.

[42] Non-systemic treatment for patients with low-volume metastatic prostate cancer - Full Text View - ClinicalTrials.gov [Internet]. Clinicaltrials.gov. 2016 [cited 15 September 2016]. Available from: https://clinicaltrials.gov/show/NCT01558427

[43] Conventional Care Versus Radioablation (Stereotactic Body Radiotherapy) for Extracranial Oligometastases - Full Text View - ClinicalTrials.gov [Internet]. Clinicaltrials.gov. 2016 [cited 15 September 2016]. Available from: https://clinicaltrials.gov/ct2/show/NCT02759783

[44] Best systemic therapy or best systemic therapy (BST) plus definitive treatment (radiation or surgery) - Full Text View - ClinicalTrials.gov [Internet]. Clinicaltrials.gov. 2016 [cited 15 September 2016]. Available from: https://clinicaltrials.gov/ct2/show/NCT01751438

[45] ISRCTN - ISRCTN15704862: testing radical prostatectomy in men with oligometastatic prostate cancer that has spread to the bone [Internet]. Isrctn.com. 2016 [cited 15 September 2016]. Available from: http://www.isrctn.com/ISRCTN15704862?q=cancer&filters=conditionCategory:Cancer&sort=&offset=9&totalResults=1796&page=1&pageSize=10&searchType=basic-search

[46] Impact of radical prostatectomy as primary treatment in patients with prostate cancer with limited bone metastases - Full Text View - ClinicalTrials.gov [Internet]. Clinicaltrials.gov. 2016 [cited 15 September 2016]. Available from: https://clinicaltrials.gov/ct2/show/NCT02454543

